# Skeletal muscle proteins involved in fatty acid transport influence fatty acid oxidation rates observed during exercise

**DOI:** 10.1007/s00424-023-02843-7

**Published:** 2023-07-18

**Authors:** Ed Maunder, Jeffrey A. Rothschild, Andreas M. Fritzen, Andreas B. Jordy, Bente Kiens, Matthew J. Brick, Warren B. Leigh, Wee-Leong Chang, Andrew E. Kilding

**Affiliations:** 1 Sports Performance Research Institute New Zealand, Auckland University of Technology, Auckland, New Zealand; 2 The August Krogh Section for Molecular Physiology, Department of Nutrition, Exercise and Sports, Faculty of Science, University of Copenhagen, Copenhagen, Denmark; 3 Department of Biomedical Sciences, Faculty of Health and Medical Sciences, University of Copenhagen, Copenhagen, Denmark; 4 Orthosports North Harbour, AUT Millennium, Auckland, New Zealand; 5 Faculty of Health and Environmental Sciences, Auckland University of Technology, Auckland, New Zealand

**Keywords:** Fat metabolism, Transporters, Cycling, Muscle

## Abstract

Several proteins are implicated in transmembrane fatty acid transport. The purpose of this study was to quantify the variation in fatty acid oxidation rates during exercise explained by skeletal muscle proteins involved in fatty acid transport. Seventeen endurance-trained males underwent a (i) fasted, incremental cycling test to estimate peak whole-body fatty acid oxidation rate (PFO), (ii) resting vastus lateralis microbiopsy, and (iii) 2 h of fed-state, moderate-intensity cycling to estimate whole-body fatty acid oxidation during fed-state exercise (FO). Bivariate correlations and stepwise linear regression models of PFO and FO during 0–30 min (early FO) and 90–120 min (late FO) of continuous cycling were constructed using muscle data. To assess the causal role of transmembrane fatty acid transport in fatty acid oxidation rates during exercise, we measured fatty acid oxidation during in vivo exercise and ex vivo contractions in wild-type and CD36 knock-out mice. We observed a novel, positive association between vastus lateralis FATP1 and PFO and replicated work reporting a positive association between FABPpm and PFO. The stepwise linear regression model of PFO retained CD36, FATP1, FATP4, and FABPpm, explaining ~87% of the variation. Models of early and late FO explained ~61 and ~65% of the variation, respectively. FATP1 and FATP4 emerged as contributors to models of PFO and FO. Mice lacking CD36 had impaired whole-body and muscle fatty acid oxidation during exercise and muscle contractions, respectively. These data suggest that substantial variation in fatty acid oxidation rates during exercise can be explained by skeletal muscle proteins involved in fatty acid transport.

**Supplementary Information:**

The online version contains supplementary material available at 10.1007/s00424-023-02843-7.

## Introduction

The capacity to oxidise fat to support energy turnover during exercise has implications for health and endurance performance [33, 38]. Plasma-derived fatty acids must undergo transmembrane transport into the muscle cell cytosol, transport in the cytosol, and then transport across the mitochondrial outer and inner membranes prior to β-oxidation. In addition to fatty acids originating from the circulation, either released from adipose tissue or from lipoprotein lipase-mediated lipoprotein triacylglycerol hydrolysis in the capillary, skeletal muscle fatty acid oxidation can be fuelled by intramuscular triacylglycerol (IMTG). IMTGs undergo lipolysis in the muscle cell cytosol before transport across the mitochondrial membranes [16]. The development of a robust capacity for fatty acid uptake and oxidation is one of the key metabolic health benefits of regular exercise [32, 38, 45].

An individual’s capacity for fatty acid oxidation has been characterised using the peak fatty acid oxidation rate (PFO) observed during fasted, incremental exercise [32]. Identification of the determinants of PFO has been the subject of considerable contemporary research, and several skeletal muscle factors have been positively associated with PFO [8]. These include type I fibre percentage, capillary density, mitochondrial proteins, and enzymes involved in β-oxidation and intramuscular triacylglycerol lipolysis [10, 37, 40, 43]. The abundance of three skeletal muscle proteins involved in the events leading to fatty acid transport into skeletal muscle and eventually the mitochondria for oxidation, a cluster of differentiation 36/SR-B3 (previously SR-B2) (CD36) [33], fatty acid binding protein 1, plasma membrane (FABPpm) [9], and carnitine palmitoyltransferase 1 (CPT1) [9], have all been positively associated with PFO. Nevertheless, a major paradigm is that fatty acid oxidation rates during exercise are mainly regulated by mitochondrial metabolic processes such as β-oxidation [22]. The role of transmembrane fatty acid transport in fatty acid oxidation during exercise is not completely understood.

**Fig. 1:**
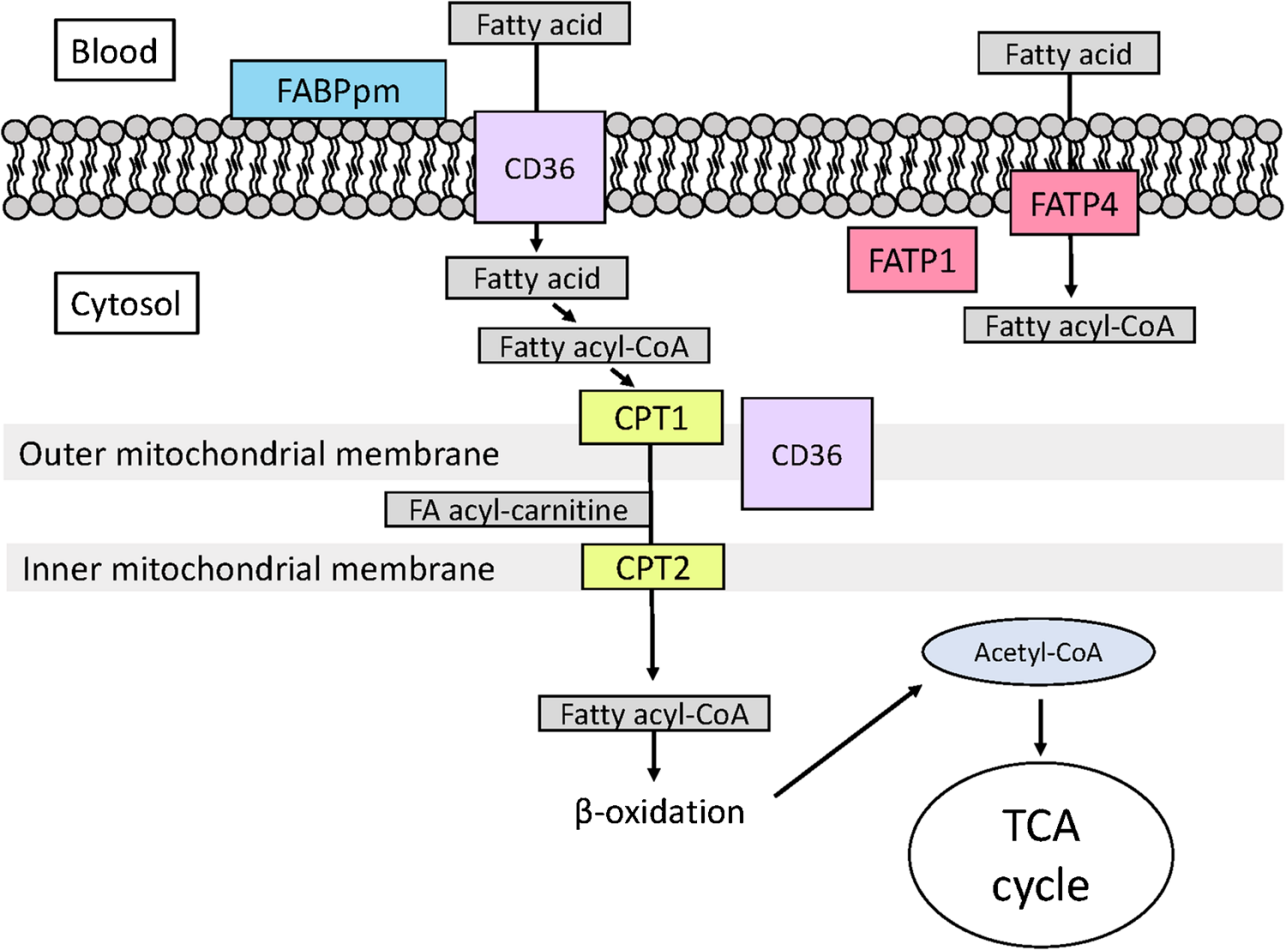
Schematic model of proteins involved in fatty acid transport in skeletal muscle. CD36, cluster of differentiation 36/SR-B3 (previously SR-B2); CPT1, carnitine palmitoyltransferase 1; CPT2, carnitine palmitoyltransferase 2; FABPpm, fatty acid binding protein plasma membrane, plasma membrane; FATP1, fatty acid transport protein 1; FATP4, fatty acid transport protein 4, FATP4; TCA, tricarboxylic acid. We acknowledge that the specific localisation of FATP1 is debated [14, 15, 48]

The skeletal muscle determinants of fatty acid oxidation during exercise may vary according to variables such as exercise intensity, duration, and feeding status, which are themselves influential determinants of substrate use [39]. For example, in our recent work, skeletal muscle CD36 content was associated with PFO but not fatty acid oxidation rate during prolonged exercise with carbohydrate feeding [33]. In this situation, fatty acid oxidation may likely be inhibited by the exogenous carbohydrates via regulatory mechanisms in the mitochondria, e.g. activation of the pyruvate dehydrogenase complex (PDC) and lowered carnitine availability [31]. This could suggest that during exercise with concomitant exogenous carbohydrate feeding, where circulating fatty acid concentrations and oxidation in muscle are likely to be low [42, 46], an individual’s propensity for fatty acid oxidation may not be dependent on the capacity for fatty acid transport across the sarcolemmal membrane, and simple diffusion may be sufficient to sustain the required fatty acid import [27]. This is supported by our observation of a strong but imperfect correlation (*r* = 0.83, 95% CI 0.57, 0.94, *P* < 0.001) between PFO and fatty acid oxidation during prolonged, fed-state exercise [33]. However, this requires further examination through the quantification of relationships between fatty acid oxidation rates during exercise and the abundance of other skeletal muscle proteins involved in fatty acid transport (Fig. 1).

To our knowledge, relationships between fatty acid oxidation rates during exercise and several skeletal muscle proteins involved in fatty acid transport, including fatty acid transport protein 1 (FATP1), fatty acid transport protein 4 (FATP4), and the enzyme carnitine palmitoyltransferase 2 (CPT2), have not been assessed. Similarly, the relative contributions made by different fatty acid transport proteins to the variation in fatty acid oxidation rates during exercise in humans have not previously been quantified, and the direct causal necessity for specific fatty acid transporters for fatty acid uptake and oxidation in skeletal muscle during exercise has only been scarcely investigated. These data have implications for furthering our understanding of the skeletal muscle determinants of fatty acid oxidation during exercise and for identifying the transport proteins that warrant most attention in studies of fatty acid metabolism during exercise.

Accordingly, the aims of the present investigation were to (i) investigate the associations between six skeletal muscle proteins, PFO, and fatty acid oxidation during fed-state exercise, (ii) use linear regression models to quantify the variation in fatty acid oxidation rates during exercise that is explained by these proteins, and (iii) identify the proteins that explain the most variation in these models. Additionally, to assess the causal role of transmembrane fatty acid transport in fatty acid oxidation rates during exercise, we measured fatty acid oxidation during in vivo exercise and ex vivo contraction protocols in wild-type (WT) and CD36 knock-out (KO) mice. We hypothesised that proteins involved in fatty acid transport would explain a large degree of variation in PFO measured during fasted, incremental cycling but explain less variation in fatty acid oxidation rates during prolonged, fed-state exercise and that mice lacking CD36 would exhibit lower fatty acid oxidation rates than wild-type controls during in vivo exercise and ex vivo contractions.

## Methods

### Ethical approval

This study was performed in accordance with the standards of the Declaration of Helsinki, 2013, and the data presented here was collected as part of a larger study published elsewhere [33, 34]. The Auckland University of Technology Ethics Committee approved all procedures in the human studies (19/146), and all participants provided written informed consent prior to participation. All animal experiments as well as the breeding protocol were approved by the Danish Animal Experimental Inspectorate and complied with the *European Convention for the Protection of Vertebrate Animals used for Experiments and other Scientific Purposes*. This study was not registered in a database. Data associated with this study are available from the corresponding author upon reasonable request.

### Human study

#### Participants

Seventeen endurance-trained male cyclists and triathletes took part in the present investigation (age, 34 ± 7 years; height, 181 ± 8 cm; body mass, 80.5 ± 9.6 kg; recent training volume, 8 ± 2 h·week^−1^; first ventilatory threshold, 206 ± 39 W; second ventilatory threshold, 265 ± 54 W; V̇O_2_peak, 4.3 ± 0.7 L·min^−1^). The present study includes pre-intervention data from a prior study of the effects of heat training on endurance performance [34] and extends findings from our recent cross-sectional study [33]. This study was conducted during the maintenance phase of training for all participants. Our dataset, in line with previous research [32], reports considerable heterogeneity in PFO (0.32–1.00 g·min^−1^), thus allowing interrogation of the role of variation in the skeletal muscle content of various proteins involved in transmembrane fatty acid transport on fatty acid oxidation rates during exercise.

#### Study design

A cross-sectional design was used in the present investigation, which has been described elsewhere [33]. Participants visited the laboratory on three occasions, ~48 h apart, for an (i) incremental cycling test after an overnight fast, (ii) resting muscle microbiopsy, and (iii) prolonged fed-state cycling assessment. The order of visits was not randomised as the incremental test data was used to define the power output during the prolonged cycling assessment.

#### Incremental cycling test

Participants arrived for the incremental cycling test at ~07:00 having fasted overnight and refrained from alcohol consumption and vigorous exercise for 24 h. Height and body mass were recorded. Cycling then commenced at 95 W, with the power output increasing by 35 W every 3 min (Excalibur Sport, Lode, Groningen, NET). Expired gases were collected throughout (TrueOne2400, ParvoMedics, Sandy, UT, USA), and the last minute at each power output was used to estimate whole-body carbohydrate (CHO) and fatty acid oxidation rates, and energy expenditure, using standard non-protein stoichiometric equations [24]. The PFO was identified as having the highest observed rate of whole-body fatty acid oxidation during the incremental cycling test [1]. A capillary blood sample was obtained from a finger at the end of each 3-min stage and analysed for blood lactate concentration (Lactate Pro 2, Arkray, Tokyo, Japan). When blood lactate concentration was > 4 mmol·L^−1^, the duration of each power output was reduced to 1 min until volitional exhaustion. The first ventilatory threshold (VT_1_) was estimated as the work rate at which the ventilatory equivalent for oxygen (V̇E·V̇O_2_^−1^) began to increase in the absence of changes in the ventilatory equivalent for carbon dioxide (V̇E·V̇CO_2_^−1^) [29]. The V̇O_2_peak was defined as the highest 15-s oxygen consumption (V̇O_2_).

#### Muscle microbiopsy

Participants arrived at the laboratory ~48 h following the incremental cycling test at ~9:00 having fasted overnight. A muscle microbiopsy was obtained from the vastus lateralis using the microbiopsy technique [17]. Local anaesthesia was applied to the skin and superficial muscle fascia, after which a microbiopsy needle was inserted into the mid-belly of the vastus lateralis to a depth of ~2 cm to recover ~15–20 mg of tissue using a spring-loaded mechanism (14G Ultimate Biopsy Needle, Zamar Care, Croatia). Muscle tissue was immediately frozen using dry ice and stored at −80 °C until further analysis.

#### Prolonged cycling assessment

Participants performed a prolonged cycling assessment ~48 h following the muscle microbiopsy, having avoided vigorous exercise in that time. They were provided with ‘base’ CHO (e.g. pasta, rice, noodles, and rolled oats) to provide 1 g·kg^−1^ for dinner at ~20:00 the evening before and 1 g·kg^−1^ for breakfast 2 h prior to the assessment to ensure that participants completed the trial in a fed-state. Participants added to these ‘base’ foods at these meals such that the total CHO intake at these two meals combined was 2.5–3.0 g·kg^−1^. Participants were provided with portable weighing scales and instructions on how to record their dietary intake at these meals. These written records were supplemented with photographs taken at the beginning and end of each meal. The trial consisted of cycling for 2 h on an ergometer (Excalibur Sport, Lode, Groningen, NET) at 80% of the power output at VT_1_ (18 °C and 60% relative humidity). Convective airflow was provided by an industrial fan (FS-75, FWL, Auckland, NZ). Participants consumed 60 g·h^−1^ of glucose in 7.5% liquid solutions at 15-min intervals throughout the constant-load phase. Expired gases were collected for 4 min every 15 min using a metabolic cart (TrueOne2400, ParvoMedics, Sandy, UT, USA), with the last 3 min of each sample used to estimate whole-body CHO and fatty acid oxidation rates using standard non-protein stoichiometric equations [24]. The mean of these values was used to represent prolonged fed-state fatty acid oxidation (FO).

#### Muscle analyses

Frozen muscle samples were rinsed and suspended to 25 mg·mL^−1^ in cold phosphate-buffered saline and ground manually using a pre-cooled glass Dounce homogeniser. Homogenate was solubilised with either extraction buffer (ab260490, Abcam®) for analysis of CD36 content or in phosphate-buffered saline (remaining analyses) to 5 mg·mL^−1^. Samples solubilised in phosphate-buffered saline were subjected to two freeze-thaw cycles prior to centrifugation to assist in the breakdown of cell membranes. Following solubilisation, samples were incubated on ice for 20 min prior to centrifugation at 16,000 g for 20 min at 4 °C.

A Bradford assay for sample protein concentration was subsequently performed in duplicate (intra-assay within-standard deviation coefficient of variation [CV], 3.7%). Briefly, a Coomassie blue G reagent was added to protein standards and samples, and optical density was measured on a spectrophotometer at 595 nm (ab102535, Abcam®). Abundances of CD36 (ab267614, Abcam®), FATP1 (MBS456856, MyBioSource), FATP4 (MBS456858, MyBioSource), CPT1 (MBS724213, MyBioSource), CPT2 (MBS7227602, MyBioSource), and FABPpm (ab222875, Abcam®) were determined via fully-quantitative enzyme-linked immunosorbent assays. These assays were performed using commercially available kits in duplicate according to the manufacturer’s instructions on a spectrophotometer (Multiskan GO, Thermo Fisher Scientific Inc., Porto Salvo, POR) and expressed relative to sample protein concentration. Achieved intra-assay CVs were 9.8%, 18.3%, 12.8%, 6.2%, 14.8%, and 14.8% for CD36, FATP1, FATP4, FABPpm, CPT1, and CPT2 abundances, respectively.

### Mouse studies

Twelve-week-old male whole-body CD36 KO mice and WT littermates were used as previously described [12, 35]. Genotyping was performed by polymerase chain reaction analysis as described [25]. Mice were housed in temperature-controlled (22 ± 1 °C) facilities, maintained on a 12 h:12 h light–dark cycle, and received standard chow (Altromin, cat. no. 1324; Brogaarden, Lynge, Denmark) and water *ad libitum*. The CD36 KO model is particularly relevant given the established relationship between PFO and CD36 in humans [33] and the observed impaired fatty acid oxidation in mice lacking CD36 [28, 35, 47].

#### In vivo treadmill exercise test with indirect calorimetry measurements

Prior to the treadmill exercise test, mice were familiarised on a treadmill apparatus (TSE Systems GmbH, Germany). Familiarisation was performed two times a day, separated by 5 h of rest for 3 days as described [13]. Thereafter, mice rested 2 days prior to the experimental day. Then, after 4 h of fasting, mice performed a maximal running speed test, which started at 10.8 m·min^−1^ at a 0% incline and increased by 2.4 m·min^−1^ every second minute until the mice were unable to keep up with treadmill speed. During this exercise test, oxygen uptake (V̇O_2_) and CO_2_ production (V̇CO_2_) were measured using a CaloSys apparatus (TSE Systems, Bad Homburg, Germany), and fatty acid oxidation rates were calculated as V̇O_2_*19kJ/l O_2_*((1-(V̇CO_2_/V̇O_2_))/0.3).

#### Palmitate uptake and oxidation in isolated rested and contracted muscle ex vivo

Contraction-stimulated exogenous palmitate oxidation in isolated soleus muscle from CD36 KO and WT mice was measured as previously described [23]. In brief, excised soleus muscles from mice fasted for 4 h and anesthetised by pentobarbital were mounted at resting tension (∼5 mN) in 15 mL vertical incubation chambers with a force transducer (Radnoti, Monrovia, CA) containing 30 °C carbogenated (95% O_2_ and 5% CO_2_) Krebs-Henseleit Ringer buffer, pH = 7.4, supplemented with 5 mM glucose, 2% fatty acid-free BSA, and 0.5 mM palmitate. After ∼20 min of preincubation, the incubation buffer was refreshed with KRB additionally containing [1–^14^C]-palmitate (0.0044 MBq·mL^−1^; Amersham BioSciences, Buckinghamshire, UK). To seal the incubation chambers, mineral oil (Cat. no. M5904, Sigma–Aldrich) was added on top. Exogenous palmitate oxidation was measured at rest and during 25-min contractions (18 trains·min^−1^, 0.6 s pulses, 30 Hz, 60 V). After incubation, incubation buffer and muscles were collected to determine the rate of palmitate oxidation as described [11, 23, 41]. Palmitate oxidation was determined as CO_2_ production (complete FA oxidation) and acid-soluble metabolites (ASM, representing incomplete FA oxidation). As no difference was observed in complete and incomplete FA oxidation between genotypes, palmitate oxidation is presented as the sum of these two forms.

Palmitate uptake was calculated as the sum of palmitate incorporation into diacylglycerol (DAG) and triacylglycerol (TG) pools and total palmitate oxidation. Palmitate incorporation into DAG and TG pools was measured as follows. From muscles that were homogenized in order to obtain the ASM, the chloroform phase was evaporated under a stream of N_2_ and re-dissolved in 50 μL of chloroform. A total of 30 μL of each sample were spotted on a silica gel plate and placed in a sealed tank containing heptane:isopropylether:acetic acid (60:40:3) for 50 min. Plates were air-dried, dipped in copper-sulphate, and heated for 15 min. DAG and TG bands were visualised by long-wave UV-light, marked, and then scraped into scintillation vials for liquid scintillation.

### Statistical analyses

Human data are expressed as the mean ± standard deviation, or with 95% confidence limits. The normality of data distributions was assessed using the Shapiro-Wilk test. Bivariate relationships were assessed using Pearson’s product-moment correlation coefficients (*r*) or Spearman’s rank-order correlation coefficients (*r*_*s*_), depending on normality, and expressed with 95% confidence limits and two-tailed *P*-values. Linear regression models of PFO and FO during the first 30 min (Early FO) and 90–120 min of fed-state cycling (Late FO) were constructed with CD36, FATP1, FATP4, CPT1, CPT2, and FABPpm abundances as inputs using the ‘lm’ function in *R*. Energy expenditure (EE) was included in the early FO and late FO models to account for the varying work rates among participants. Models were then put through a stepwise model selection process involving forward inclusion and backward elimination using 500 bootstrap resamples [3] to identify the most predictive and parsimonious combination of variables through the Akaike information criterion (AIC) using the ‘boot.stepAIC’ function from the ‘bootStepAIC’ R package. Model assumptions were checked using the ‘performance’ *R* package. Standardised regression coefficients were calculated for all models using the ‘Parameters’ *R* package. A series of linear mixed models were used to estimate differences between the WT and CD36 KO mice for fatty acid oxidation rate during an in vivo maximal treadmill exercise test and palmitate uptake and oxidation during muscle contractions. Time point (each minute during the maximal test, or at rest and during muscle contraction) and the group were specified as fixed effects, with the animal ID specified as a random intercept. Contrasts between each group and time point were estimated using the emmeans R package, with multiple comparisons adjusted using the Holm correction. All analysis was performed in *R* (version 4.1.2) with RStudio (version 2022.07.1+554). Raw data is available at this link: https://osf.io/4yqdt/. Statistical significance was inferred when *P* ≤ 0.05.

## Results

### Human study

**Fig. 2:**
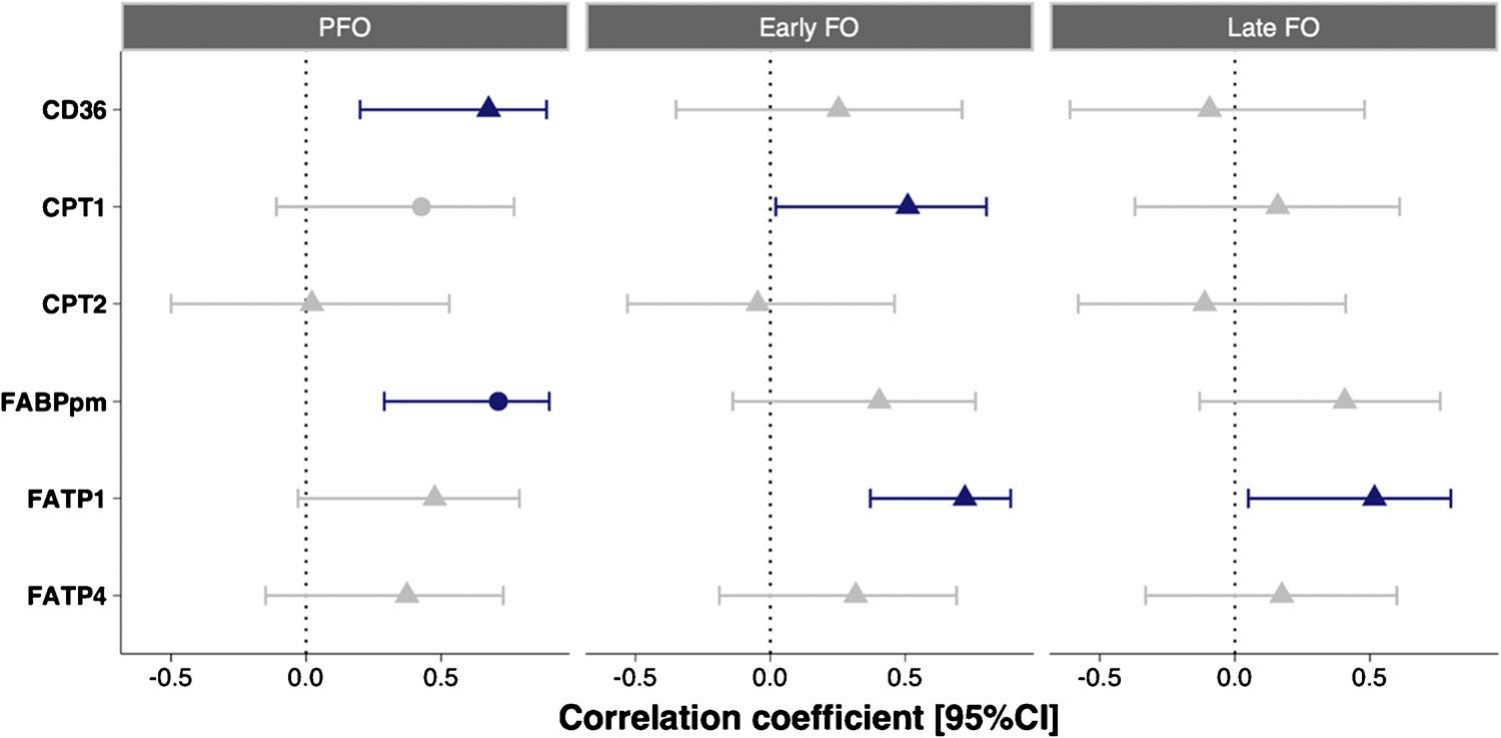
Bivariate correlations between the peak fatty acid oxidation rate (PFO) measured during fasted, incremental cycling, fatty acid oxidation during the first 30 min of prolonged, fed-state cycling (early FO), and 90–120 min of prolonged, fed-state cycling (late FO) and vastus lateralis carnitine palmitoyltransferase 1 (CPT1), carnitine palmitoyltransferase 2 (CPT2), fatty acid binding protein 1 (FABPpm), cluster of differentiation 36 (CD36), fatty acid transporter 1 (FATP1), and fatty acid transport 4 (FATP4). Circles denote Pearson’s correlation coefficient, and triangles ‘ρ’ denote Spearman’s rank-order correlation coefficient, with 95% confidence intervals shown by the solid lines. Colours denote statistical significance (*P* < 0.05)

**Fig. 3:**
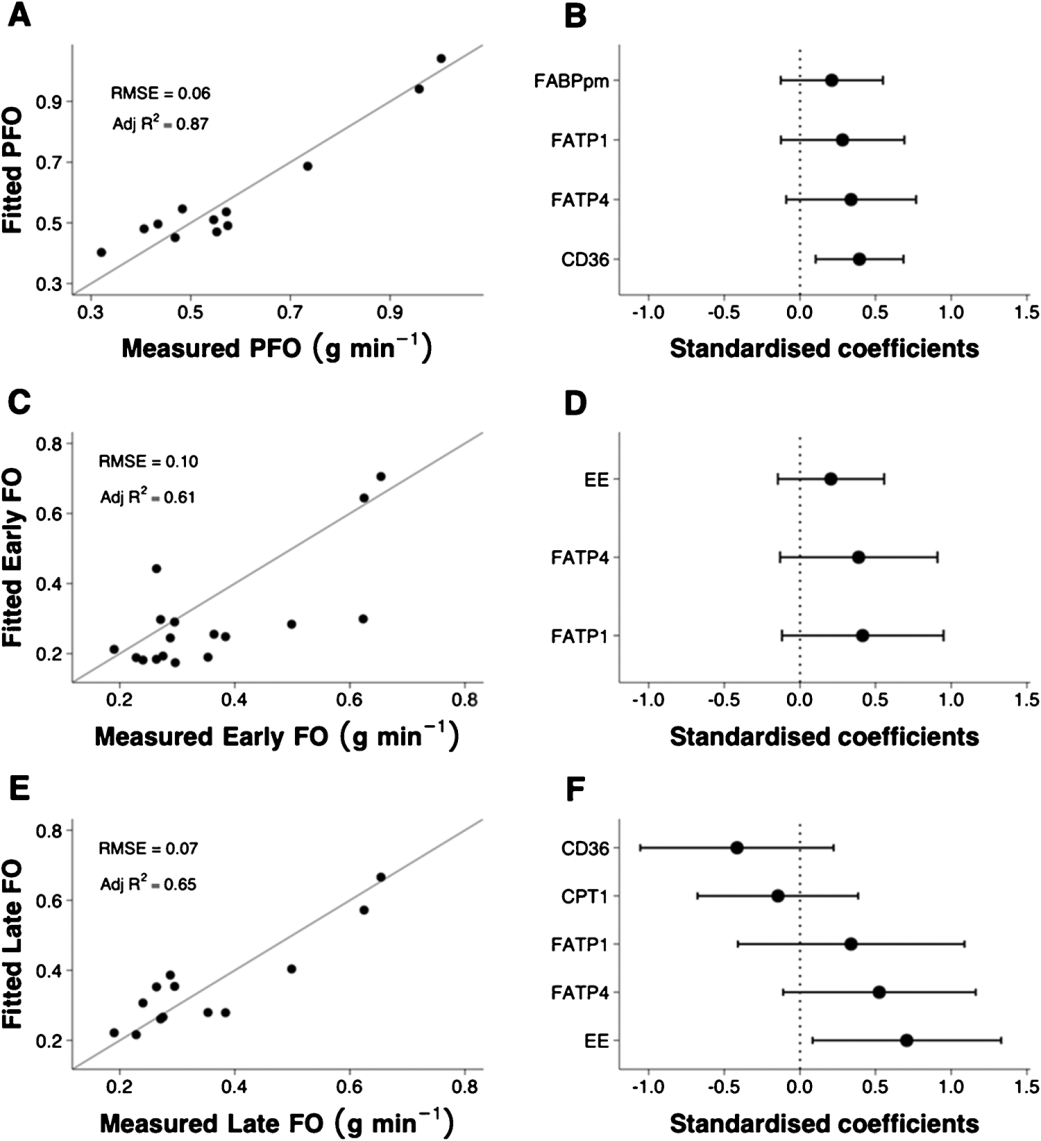
Modelling analysis with stepwise selection for peak fatty acid oxidation rate during fasted, incremental cycling (PFO), mean fatty acid oxidation during the first 30 min of prolonged, fed-state cycling (early FO), and mean fatty acid oxidation during 90–120 min of prolonged, fed-state cycling (late FO). Fitted model values compared with measured values are shown in (**A**), (**C**), and (**E**). Standardised coefficients with 95% confidence intervals are shown in (**B**), (**D**), and (**F**). Adj *R*^2^, Adjusted *R*^2^ value; CD36, cluster of differentiation 36/SR-B3 (previously SR-B2); CPT1, carnitine palmitoyltransferase 1; EE, energy expenditure; FABPpm, fatty acid binding protein plasma membrane; FATP1, fatty acid transport protein 1; FATP4, fatty acid transport protein 4; RMSE, root mean square error

Positive associations were observed between specific proteins and both PFO and FO (Fig. 2, Supplementary Figure 1,2,3). Linear regression identified that skeletal muscle content of the studied proteins explained ~87% of PFO, and these proteins in addition to whole-body EE explained ~61% and 65% of the variation in early and late FO, respectively (Table 1 and Fig. 3).

**Table 1:** Linear regression models of peak fatty acid oxidation rate (PFO) measured during fasted, incremental cycling, mean fatty acid oxidation rate during the first 30 min of prolonged, fed-state cycling (early FO), and mean fatty acid oxidation rate during 90–120 min of prolonged, fed-state cycling (late FO)

Outcome	Number of observations^a^	Adjusted *R*^2^	Input variable	Standardised coefficient
PFO	12	0.87	CD36	0.39 95% CI [0.10, 0.68]
			FATP1	0.28 95% CI [−0.13, 0.69]
			FATP4	0.34 95% CI [−0.09, 0.77]
			FABPpm	0.21 95% CI [−0.13, 0.55]
Early FO	17	0.61	FATP1	0.42 95% CI [−0.12, 0.95]
			FATP4	0.39 95% CI [−0.13, 0.91]
			EE	0.21 95% CI [−0.15, 0.56]
Late FO	13	0.65	CD36	−0.42 95% CI [−1.06, 0.22]
			FATP1	0.34 95% CI [−0.41, 1.09]
			FATP4	0.53 95% CI [−0.11, 1.16]
			CPT1	−0.15 95% CI [−0.68, 0.39]
			EE	0.71 95% CI [0.08, 1.33]

^a^Number of observations per model varies due to missing values for some markers. Abbreviations: *CD36*, cluster of differentiation 36/SR-B3 (previously SR-B2); *CPT1*, carnitine palmitoyltransferase 1; *CPT2*, carnitine palmitoyltransferase 2; *EE*, energy expenditure; *FABPpm*, fatty acid binding protein plasma membrane; *FATP1*, fatty acid transport protein 1; *FATP4*, fatty acid transport protein 4. For each model, only the variables retained in the stepwise selection process are included and shown. Adjusted *R*^2^ shows the fit of each model to the data; the standardised coefficient allows comparisons to be made between input variables using the same units

### Mouse studies

**Fig. 4:**
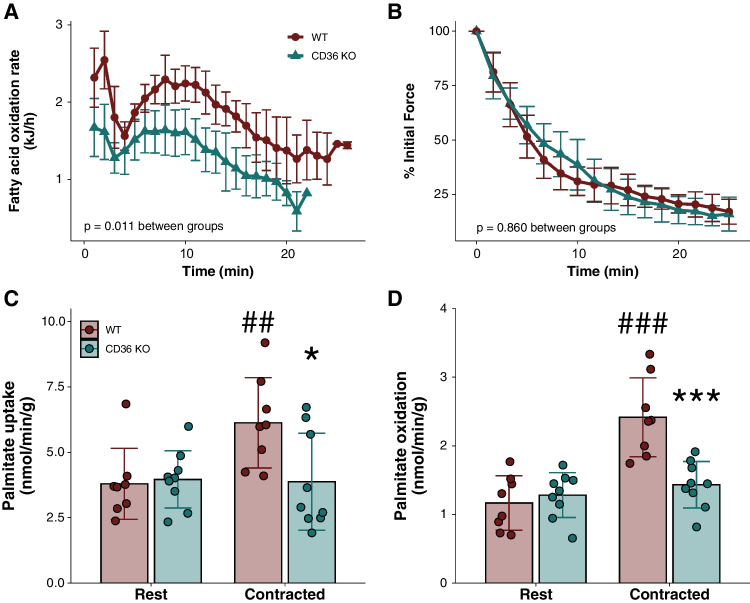
**A** Fatty acid oxidation rates in 12-week-old male CD36 knock-out (KO) mice and wild type (WT) littermates during a maximal treadmill running test. WT *n* = 4 and CD36 KO *n* = 6. **B**–**D** Contraction force (% of initial value) (**B**), palmitate uptake (**C**), and palmitate oxidation (**D**) in soleus muscle from 12-week-old male CD36 knock-out (KO) mice and wild type (WT) littermates at rest and during muscle contractions ex vivo. WT *n* = 8 and CD36 KO *n* = 9. ##, ###Significantly different (*P* < 0.01/*P* < 0.001) from rest within WT. *,***Significantly different (*P* < 0.05/*P* < 0.001) from WT within contracted. Data are mean ± SD. CD36, cluster of differentiation 36/SR-B3 (previously SR-B2)

To further verify the causal role of fatty acid transport protein content in the regulation of substrate oxidation during exercise, we took advantage of mice lacking the fatty acid transporter CD36. In these mice, we observed lower FA oxidation during an in vivo maximal treadmill exercise test compared with control wild-type mice (*P* = 0.011), suggesting a lower ability to oxidise fatty acids during exercise in mice lacking CD36 (Fig. 4A). Importantly, these mice are whole-body KO mice (constitutive KO) [12]. Therefore, to directly investigate the importance of CD36 in fatty acid oxidation in skeletal muscle during muscle contractions, we took a novel approach in this mouse model and utilised an ex vivo incubation system measuring palmitate oxidation during muscle contractions. Force production was similar between soleus muscles from WT and CD36 KO mice (Fig. 4B). In line with the findings during in vivo running, we observed that soleus muscles from WT mice had increased palmitate uptake and oxidation during muscle contractions by 34% and 120%, respectively, compared with rest, which was completely abolished in mice lacking CD36 (Fig. 4C, D).

## Discussion

The primary aims of the present study were to investigate the associations between the skeletal muscle content of various proteins involved in fatty acid transport and a ‘capacity’ measure of exercise fatty acid oxidation rates (PFO) and a ‘fuel selection’ measure during prolonged cycling in the fed state (FO) and to quantify the variation in fatty acid oxidation rates explained by skeletal muscle fatty acid transport protein content using linear regression. Our primary findings were that (i) skeletal muscle FATP1 content was positively associated with PFO and FO thereby emerging as a novel factor in the regulation of skeletal muscle fatty acid oxidation during exercise, (ii) a substantial degree of variation in PFO (~87%) and fatty acid oxidation during the early (~61%) and later (~65%) phase of a bout of prolonged, fed-state cycling could be explained by linear regression models containing fatty acid transport proteins, (iii) FATP1 and FATP4 emerged as contributors to models of both PFO and FO, and (iv) in mice lacking CD36, whole-body fatty acid oxidation during in vivo exercise was markedly lowered compared with wild-type mice, and both increased palmitate uptake and oxidation during ex vivo muscle contractions were completely abolished in mice lacking CD36, suggesting that CD36 is important for FA uptake during muscle contractions/exercise. Altogether, these data have implications for our understanding of the skeletal muscle determinants of fatty acid oxidation rates during exercise and indicate that skeletal muscle proteins involved in fatty acid transmembrane transport influence fatty acid oxidation rates during exercise.

Our observation that vastus lateralis FATP1 content was positively associated with PFO (*r*_*s*_ = 0.48 [−0.03, 0.79], *P* = 0.06) suggests FATP1 may be a protein of importance for fatty acid uptake and oxidation (likely indirectly via acyl-CoA synthetase activity or cytosolic transport) in human skeletal muscle during exercise. This is in line with findings in rodents, in which overexpression of FATP1 in rat and mouse muscle increased fatty acid transport across the sarcolemma and increased fatty acid oxidation at rest [18, 36]. On the other hand, the rate of fatty acid transport into giant vesicles, derived from red and white rat skeletal muscle, was highly correlated with the sarcolemmal content of CD36 and FABPpm but not FATP1 during resting conditions [20, 30]. Together, this emphasises that whereas FATP1 might not be necessary, but sufficient, for fatty acid uptake in rodent muscle at rest, it seems to play a significant role in human skeletal muscle during exercise. We previously observed that vastus lateralis CD36 protein content was positively associated with PFO (reported here in Fig. 2). These results may demonstrate that during exercise in the fasted state, when circulating fatty acid concentrations and rates of appearance are substantial [42, 46], an individual’s capacity for fatty acid oxidation may be at least somewhat dependent on their ability to transport fatty acids from the circulation into the muscle cell cytosol. This conclusion is supported by a previous study reporting a positive association between PFO and vastus lateralis FABPpm protein content [9], a superficial membrane-located protein which is also implicated in fatty acid transport [7] and was replicated here (*r* = 0.71, [0.29, 0.90], *P* = 0.004). In addition, our stepwise linear regression model of PFO retaining four proteins involved in fatty acid binding and transport (FABPpm, CD36, FATP1, and FATP4) explained ~87% of the variation. Collectively, these findings challenge the view that fatty acid oxidation during exercise is mainly dependent on mitochondrial biochemical regulatory mechanisms [22] and indicate that the protein-mediated uptake of fatty acids across the sarcolemma is important.

To further clarify the causal importance of transmembrane fatty acid transport as a rate-limiting and regulatory step in the capacity for skeletal muscle fatty acid oxidation during exercise, we followed up the associative evidence in humans with direct genetic manipulations in mice. We found that in mice lacking CD36, whole-body fatty acid oxidation during in vivo exercise was markedly lowered compared with wild-type mice (Fig. 4). This is in line with previous findings of impaired fatty acid oxidation in mice lacking CD36 on a whole-body level [19, 28, 35, 47], the observed exercise-induced sarcolemmal CD36 translocation in both rat and human muscle [7], and observations of increased fatty acid oxidation alongside increased CD36 following exercise training and CD36 overexpression [35, 44]. Importantly, however, a lack of CD36 on a whole-body level could affect fatty acid oxidation rates in skeletal muscle indirectly through effects in adipose tissue or endothelial cells. Moreover, previous attempts with overexpression of CD36 in skeletal muscle suggest that CD36 is sufficient to increase muscle fatty acid oxidation. However, to directly investigate whether CD36 is necessary for fatty acid uptake and oxidation specifically in muscle, we subjected isolated muscles from mice lacking CD36 to ex vivo contractions. Hereby, we could further show that increased palmitate uptake and oxidation during ex vivo muscle contractions were completely abolished in mice lacking CD36 (Fig. 4), which is the first data to show that CD36 specifically in muscle is directly necessary for palmitate uptake and oxidation during muscle contractions. These data align with and extend upon previous observations of blunted effects of caffeine and 5-aminoimidazole-4-carboxamide-1-β-D-ribofuranosidon (AICAR) on fatty acid transport and oxidation in mouse muscle lacking CD36 [6, 28]. Collectively, the data presented here and previously provide solid evidence that fatty acid transmembrane transport is of great importance for the capacity to oxidise fatty acids in skeletal muscle during exercise and that CD36 is an important player in this.

Our measures of FO do not indicate an individual’s capacity for whole-body fatty acid oxidation but instead their propensity for fatty acid oxidation under specific circumstances, that is, with prior and concurrent carbohydrate feeding and at a fixed, moderate-intensity work rate. The moderate relationships between FATP1 and early and late FO seem to refute our previous suggestion that an individual’s propensity for fatty acid oxidation during exercise in the fed state may be unrelated to their capacity to translocate fatty acids across the sarcolemmal membrane [33]. Indeed, stepwise linear regression models constructed using only the content of the vastus lateralis fatty acid transport proteins studied predicted ~61–65% of the variation in FO, albeit with whole-body EE included within these models to account for differences in external work rates (Table 1). Whilst this appears lower than variation in PFO explained by fatty acid transporter proteins (~87%), these data do support trans-membrane fatty acid transport capacity as a determinant of fatty acid oxidation rates during exercise in the fed state.

The vastus lateralis content of the enzymes CPT1 and CPT2, which are involved in the biochemical reactions by which fatty acyl-CoA is translocated across the outer and inner mitochondrial membranes [2, 16], displayed minimal associations with PFO or FO (Fig. 1). The vastus lateralis CPT1 protein content has previously been positively associated with PFO [9], although we only found a significant correlation between CPT1 and early FO (Fig. 2). These data may suggest that an individual’s propensity for fatty acid oxidation during exercise may be more related to their ability to translocate fatty acids across the membrane into the cytosol than from the cytosol across the mitochondrial membranes. Importantly, this does not exclude the potential for the mitochondrial regulatory mechanism to be crucial, especially in the fine tuning of substrate selection for oxidation in skeletal muscle during exercise, e.g. when fatty acid oxidation is lowered and dependency on carbohydrate oxidation increases when changing from moderate to high-intensity exercise [22]. A useful progression of our data would be to interrogate changes to the skeletal muscle protein content of sarcolemmal and mitochondrial membrane fatty acid transporters in response to an exercise training intervention and investigate how those changes relate to fatty acid oxidation rates during exercise after training. Our regression analyses could be repeated in this context to discern if the proteins contributing the most variation to fatty acid oxidation rates change. Previous work has, for example, observed increased FATP4 and decreased FATP1 protein content following training concomitant with increased fatty acid oxidation rates [21].

A single, dominant contributor to models of PFO and FO could not be identified. The proteins FATP1 and FATP4 were retained after stepwise selection in all models, whilst CD36 and FABPpm were retained as positive contributors in the model of PFO, and CD36 and CPT1 were retained in the model of late FO. The substantial variation explained by the models, compared to the more modest strength of the simple bivariate correlations (Fig. 2), appears to indicate that multiple fatty acid transport proteins should be assessed by researchers seeking to characterise the capacity for fatty acid oxidation at the skeletal muscle level and that fatty acid oxidation rates are determined at multiple regulatory steps rather than by a single, rate-limiting point. This aligns with rodent muscle data reporting differential roles for the different transport proteins in transmembrane fatty acid transport and regulation of fatty acid oxidation [36]. The data presented here may provide insight into the most appropriate proteins to assess in these circumstances.

Fatty acid oxidation can be influenced by a number of variables including exercise duration and intensity, pre- and peri-exercise nutrition intake, and habitual diet [26, 39]. Thus, it is likely that the physiological determinants and limiting steps may differ between PFO and FO during continuous exercise. While PFO can be thought of as a capacity measurement (i.e., the peak rate at which fatty acids can be oxidised), FO during extended exercise can be considered a regulatory measurement (i.e., how much fatty acid is oxidised in each context). Furthermore, due to changes in muscle glycogen and cellular energy homeostasis during extended exercise, it is possible that key determinants of fatty acid metabolism change throughout exercise. However, this requires further investigation. To obtain a more complete picture of the determinants of fatty acid oxidation during various stages of extended exercise, future research could explore relationships between the proteins involved in fatty acid binding and transport, changes in muscle glycogen during exercise, and other factors thought to be involved in fatty acid oxidation during exercise including the fatty acyl-CoA/malonyl-CoA ratio, muscle pH, and enzymes in the β-oxidation pathway [14, 16].

Future research should repeat these analyses in female populations, given that substrate utilisation profiles and expression of fatty acid transport proteins in skeletal muscle appear to differ between sexes [4]. Similarly, these analyses could also be repeated in metabolically unhealthy individuals to provide insight into the pathophysiology of metabolic disease states, given that disordered fatty acid metabolism is implicated in the development of skeletal muscle insulin resistance [5].

In summary, regression models constructed using *vastus lateralis* fatty acid binding and transport proteins were able to explain a substantial degree of variation in PFO measured during fasted, incremental cycling (~87%), as well as the propensity for fatty acid oxidation during prolonged, fed-state cycling when models were constructed with whole-body EE included (~61–65%). The skeletal muscle content of FATP1 emerged as a novel factor associated with PFO. Together with our additional findings of impaired fatty acid oxidation during in vivo exercise and more importantly during ex vivo contractions in isolated muscle in mice lacking the fatty acid transport protein CD36 and similar observations made in previous work [19, 28, 35, 47], our data provide further support for the contention that the transmembrane protein CD36 may be an important determinant of an individual’s propensity for fatty acid oxidation during exercise. These data add to our growing understanding of the skeletal muscle factors that determine fatty acid oxidation during exercise. 

## Supplementary information

ESM 1

ESM 2

ESM 3

#### Author contribution

E.M. and A.E.K. conceived and designed human research. E.M., W.L.C., M.J.B., and W.B.L. conducted human experiments and collected the data. A.M.F., A.B.J., and B.K. conceived, designed, and conducted the animal research. E.M. and J.A.R. analysed the data. E.M. and J.A.R. drafted the manuscript. All authors read, revised, and approved the manuscript.

## Data Availability

Data and code are available from this link: https://osf.io/4yqdt/.

## Abbreviations

ASM: Acid-soluble metabolites
CD36: Cluster of differentiation 36/SR-B3
CPT1: Carnitine palmitoyltransferase 1
CPT2: Carnitine palmitoyltransferase 2
DAG: Diacylglycerol
FABPpm: Fatty acid binding protein plasma membrane
FATP1: Fatty acid transport protein 1
FATP4: Fatty acid transport protein 4
FO: Fatty acid oxidation rate
IMTG: Intramuscular triacylglycerol
PDC: Pyruvate dehydrogenase complex
PFO: Peak fatty acid oxidation rate
TG: Triacylglycerol
VLDL-TG: Very-low density lipoprotein triacylglycerol
VT_1_: First ventilatory threshold
WT: Wild type

## Code availability

Code is available using this link: https://osf.io/4yqdt/.
