# Supplementary figures and images for: Skeletal muscle proteins involved in fatty acid transport influence fatty acid oxidation rates observed during exercise
Source: Pflugers Arch. 2023 Jul 18;475(9):1061–72. doi: 10.1007/s00424-023-02843-7 (PMC10409849; doi:10.1007/s00424-023-02843-7)

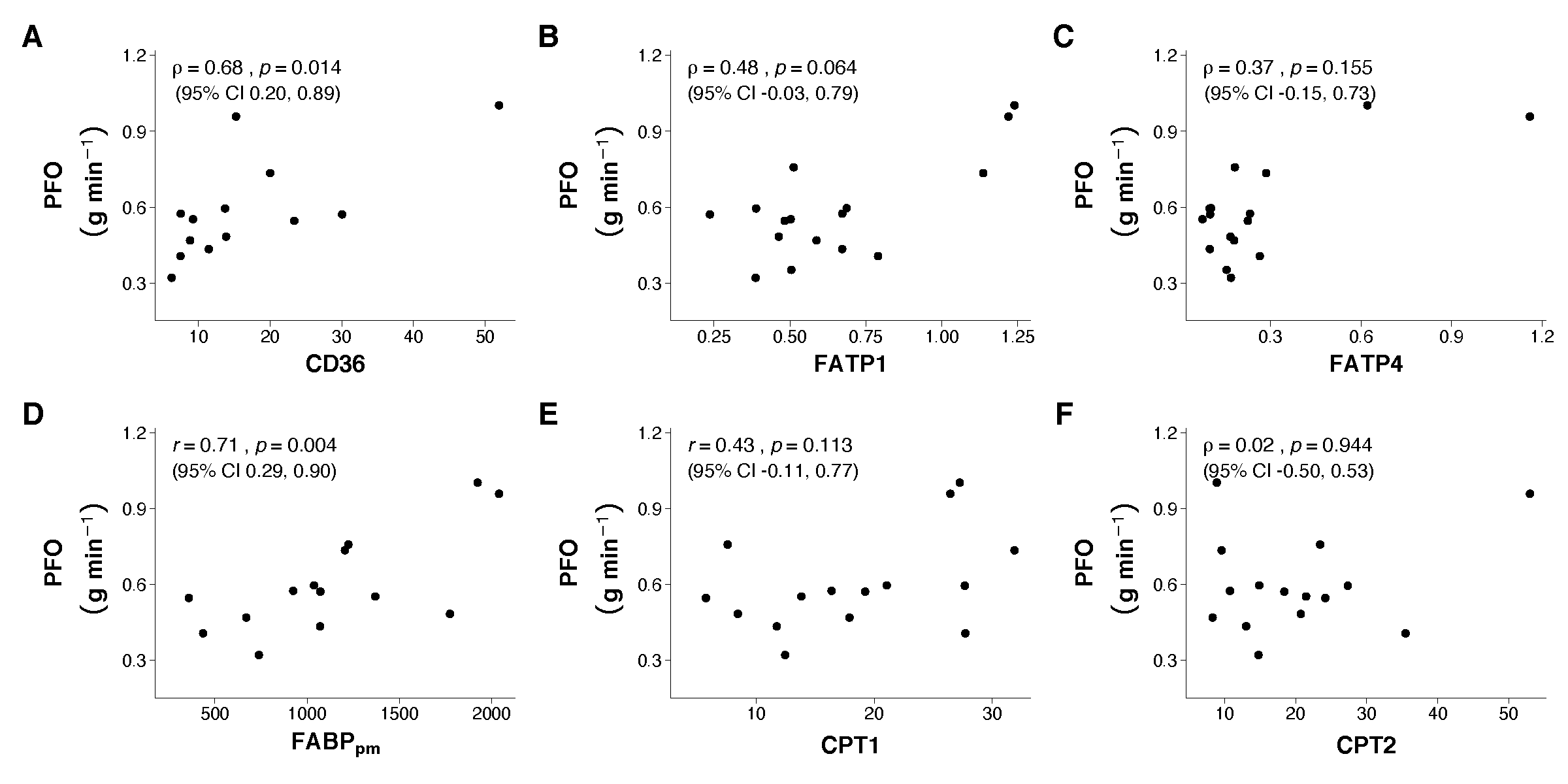

Supplement: Supplementary file 1 — ESM 1 [file 424_2023_2843_MOESM1_ESM.png]

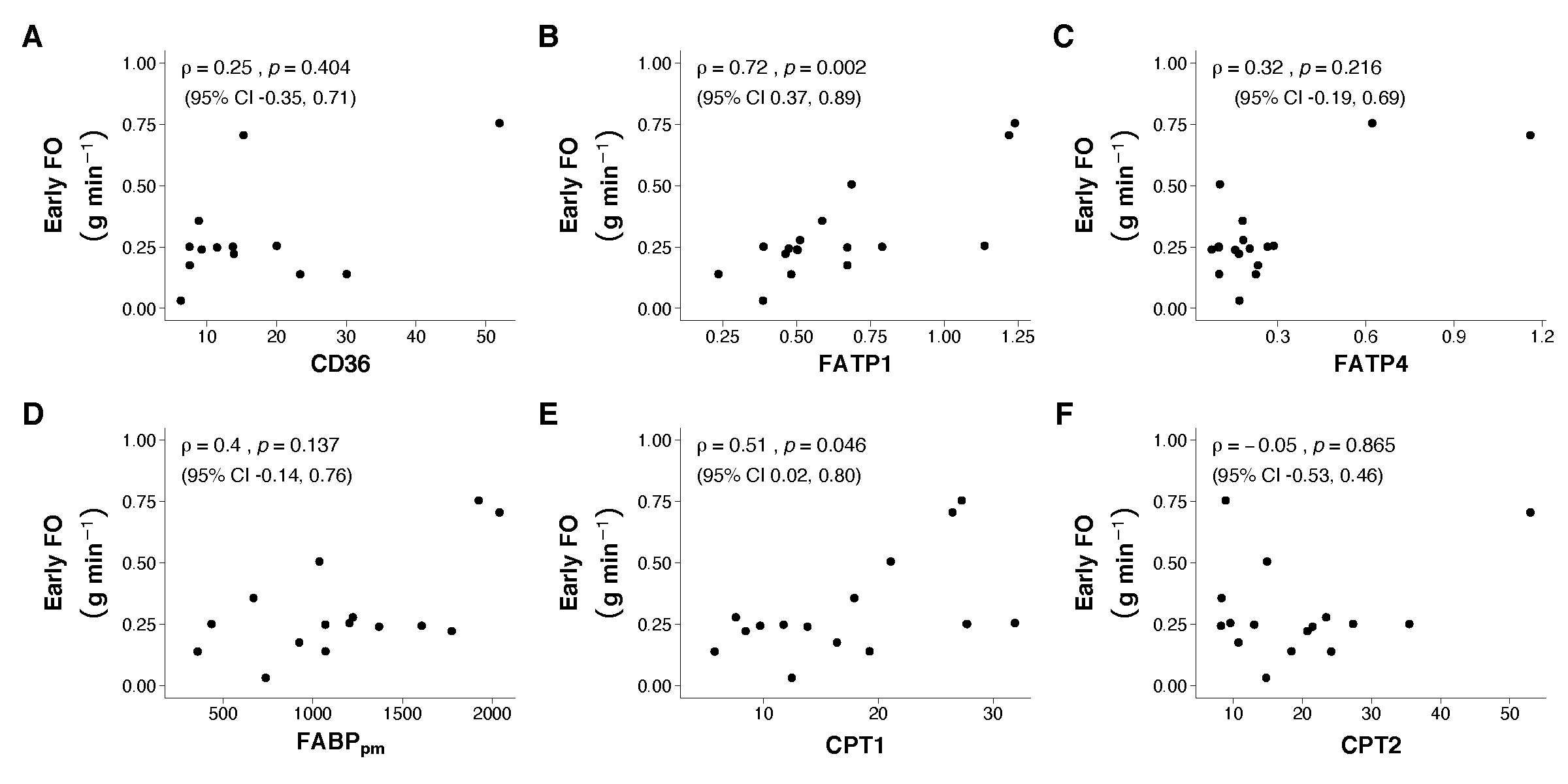

Supplement: Supplementary file 2 — ESM 2 [file 424_2023_2843_MOESM2_ESM.png]

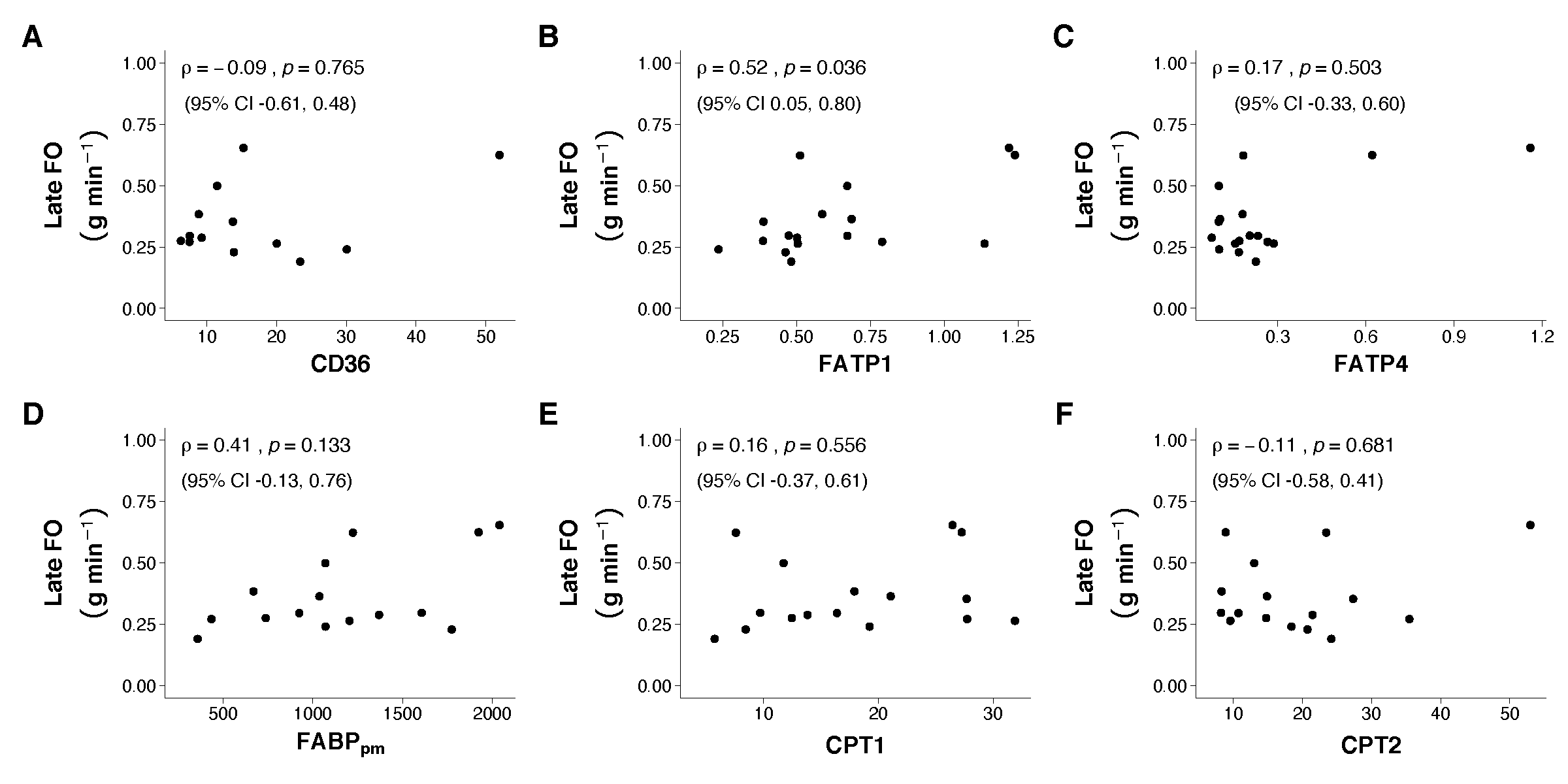

Supplement: Supplementary file 3 — ESM 3 [file 424_2023_2843_MOESM3_ESM.png]
